# Targeting hippocampal hyperactivity with real-time fMRI neurofeedback: protocol of a single-blind randomized controlled trial in mild cognitive impairment

**DOI:** 10.1186/s12888-021-03091-8

**Published:** 2021-02-09

**Authors:** Katharina Klink, Urs Jaun, Andrea Federspiel, Marina Wunderlin, Charlotte E. Teunissen, Claus Kiefer, Roland Wiest, Frank Scharnowski, Ronald Sladky, Amelie Haugg, Lydia Hellrung, Jessica Peter

**Affiliations:** 1 University Hospital of Old Age Psychiatry and Psychotherapy, Bern University, Bern, Switzerland; 2 Department of Technology and Innovation, Inselgruppe AG, Bern University, Bern, Switzerland; 3 Translational Research Center, University Hospital of Psychiatry and Psychotherapy, Bern University, Bern, Switzerland; 4 Neurochemistry Laboratory, Department of Clinical Chemistry, Amsterdam Vrije University, Amsterdam, The Netherlands; 5 Support Center for Advanced Neuroimaging, Institute for Diagnostic and Interventional Neuroradiology, Inselspital, Bern University, Bern, Switzerland; 6 Department of Basic Psychological Research and Research Methods, Vienna University, Vienna, Austria; 7 Department of Psychiatry, Psychotherapy, and Psychosomatics, Zurich University, Zurich, Switzerland; 8 Department of Economics, Zurich University, Zurich, Switzerland

**Keywords:** Hippocampal hyperactivity, Real-time fMRI, Neurofeedback, Pattern separation, Episodic memory

## Abstract

**Background:**

Several fMRI studies found hyperactivity in the hippocampus during pattern separation tasks in patients with Mild Cognitive Impairment (MCI; a prodromal stage of Alzheimer’s disease). This was associated with memory deficits, subsequent cognitive decline, and faster clinical progression. A reduction of hippocampal hyperactivity with an antiepileptic drug improved memory performance. Pharmacological interventions, however, entail the risk of side effects. An alternative approach may be real-time fMRI neurofeedback, during which individuals learn to control region-specific brain activity. In the current project we aim to test the potential of neurofeedback to reduce hippocampal hyperactivity and thereby improve memory performance.

**Methods:**

In a single-blind parallel-group study, we will randomize *n* = 84 individuals (*n* = 42 patients with MCI, *n* = 42 healthy elderly volunteers) to one of two groups receiving feedback from either the hippocampus or a functionally independent region. Percent signal change of the hemodynamic response within the respective target region will be displayed to the participant with a thermometer icon. We hypothesize that only feedback from the hippocampus will decrease hippocampal hyperactivity during pattern separation and thereby improve memory performance.

**Discussion:**

Results of this study will reveal whether real-time fMRI neurofeedback is able to reduce hippocampal hyperactivity and thereby improve memory performance. In addition, the results of this study may identify predictors of successful neurofeedback as well as the most successful regulation strategies.

**Trial registration:**

The study has been registered with clinicaltrials.gov on the 16th of July 2019 (trial identifier: NCT04020744).

## Background

Dementia due to Alzheimer’s disease (AD) is a neurodegenerative disorder associated with cognitive and functional decline. The underlying pathological process begins at least a decade before any clinical symptoms [1] and is characterized by neuronal cell death, extracellular deposits of amyloid-ß (Aβ) and intracellular formation of fibrillary aggregates of abnormally phosphorylated tau [2]. Activity in the hippocampus, as measured by functional magnetic resonance imaging (fMRI), is among the key emerging neuroimaging markers that allow an improved AD risk prediction [3]. In AD, hippocampal activity is typically decreased during memory tasks due to hippocampal atrophy. However, several fMRI studies in the prodromal stage of AD (i.e., in Mild Cognitive Impairment, MCI) have found increased hippocampal activity during memory tasks that was associated with memory deficits, subsequent cognitive decline and faster clinical progression [4–6]. Pattern separation, a process thought to critically depend on the hippocampus, has been particularly used as a task to show increased hippocampal activity in patients with MCI [7, 8]. Pharmacological treatment of hyperactivity in patients with MCI, or in mice with increased levels of Aβ, significantly reduced activity in the hippocampus and improved memory performance in pattern separation tasks [7]. Although one may expect that reducing brain activation in a given area will lead to a drop in performance, there is emerging evidence that hyperactivity in the hippocampus has a negative, rather than a positive, impact on cognition [9]. Thus, reducing excess hippocampal activity may present a promising therapeutic target. Pharmacological interventions, however, are prone to side effects such as headache, diarrhoea, or sleep disturbances. In addition, an elderly population is likely to be on other medication so - apart from the possible side effects - drug interaction may be a problem. An alternative approach may be real-time fMRI neurofeedback. With real-time fMRI neurofeedback, participants train to voluntarily ‘control’ region specific brain activity [10, 11]. The training is accomplished by continuously measuring brain activity in real-time, and providing feedback to the participant about the ongoing activity in the targeted brain area [11].

The current study aims to test whether real-time fMRI neurofeedback is capable of reducing hippocampal hyperactivity in patients with MCI and, in addition, whether the reduction of hyperactivity will be associated with an improvement in memory performance. Comparable to previous studies, we will deploy pattern separation tasks to assess hippocampal activity as well as memory performance (e.g., [7, 8]). As there is evidence for an association between Aβ and hippocampal hyperactivity [9], we will determine Aβ levels in blood. In addition, we will examine variables that may predict neurofeedback success and identify the most successful regulation strategies.

## Methods

### Participants eligibility and recruitment

Eligible participants will be between 60 and 80 years of age, fluent in German, with normal or corrected-to-normal vision. Patients with MCI will be included if their memory performance is below age-, gender-, and education-adjusted norms as assessed with the delayed recall score of a list of words included in the CERAD neuropsychological battery (CERAD [12];). Other cognitive functions may also be below the norm but an impairment in memory performance is mandatory since this subgroup is particularly prone to hippocampal hyperactivity [7]. In addition, they will need to a) report a cognitive complaint, b) show no impairment in activities of daily living, c) show no dementia but d) show signs of neuronal injury (i.e., atrophy in the hippocampus or the medial temporal lobe) according to updated criteria [13]. This will provide intermediate certainty that MCI will develop into AD [13].

Healthy elderly participants will be included if their Montreal Cognitive Assessment (MoCA) score is ≥26 [12] and their Geriatric Depression Score (GDS) is ≤5 [14]. All participants will need to give written informed consent before the study. Exclusion criteria would be major psychiatric, neurological, or medical disorders or a history of epilepsy or severe head injury, current or life-time substance abuse, contra-indications to MRI, or psychoactive medication.

We will recruit healthy elderly volunteers via newspaper advertisements. Patients with MCI due to AD pathology will be recruited from the local memory clinic where they will receive their diagnosis. The study has been approved by the Ethics Committee of the Canton of Bern (2019–00958) and will be conducted in accordance with the declaration of Helsinki. Recruitment will continue until the required number of participants is reached.

### Screening assessment and group allocation

We will screen participants during a telephone call (and only invite them to participate in the study if deemed eligible). Those who will meet inclusion criteria will be randomly assigned to the experimental group or the control group. The experimental group will downregulate activity in the hippocampus; the control group will downregulate activity in the intraparietal sulcus, a region that plays a key role in spatial attention [15] but not in pattern separation or memory. The participants will not be informed which area they will need to regulate (i.e., from which area they will receive feedback). Thus, we will apply a single-blind, randomized, parallel-group design. Choosing an alternative region in a control group will ensure physiological specificity to ensure that only the regulation of hippocampal activity (and not the regulation of any other brain region) will lead to a reduction of hippocampal hyperactivity and, in consequence, to an improvement in memory performance [16]. Group allocation will be conducted by the Clinical Trials Unit Bern with computer-generated random numbers, stratified for age, gender, and group status (i.e., MCI or healthy volunteers).

### Study procedure

**Fig. 1:**
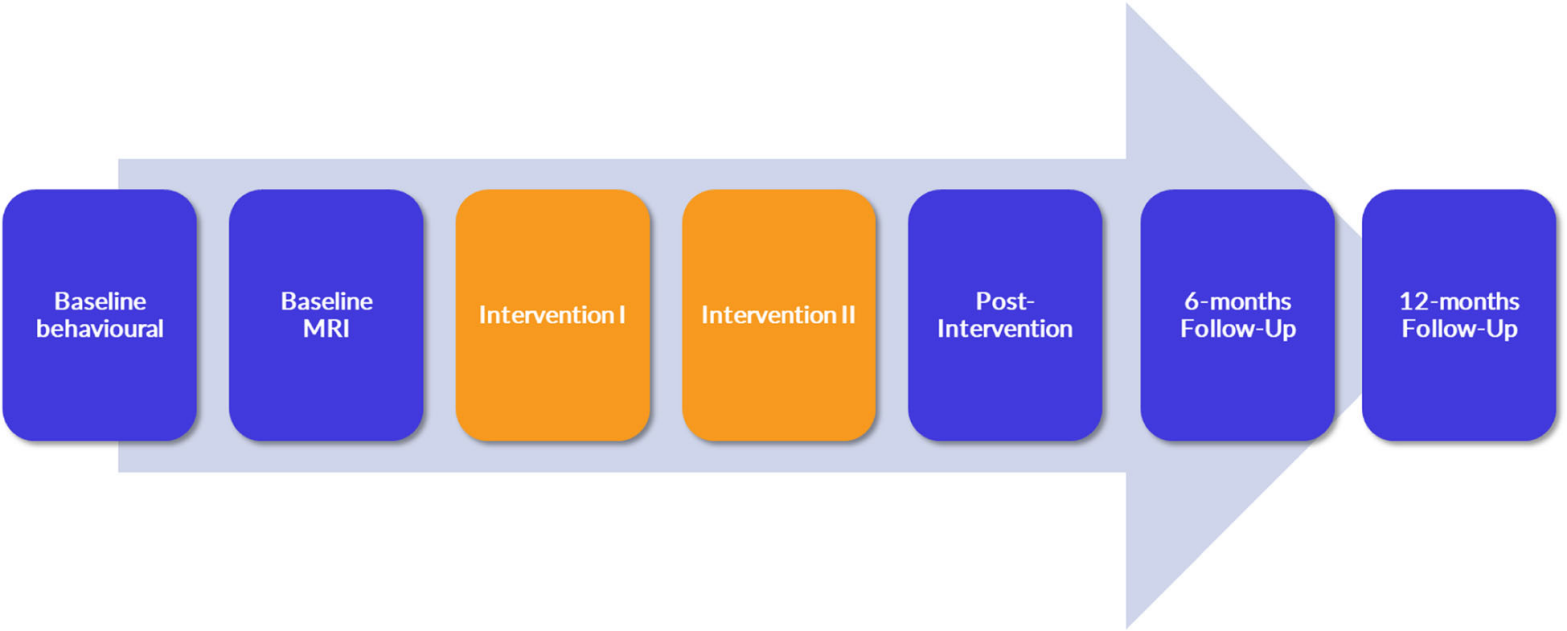
Study procedure. We will include *n* = 84 participants (*n* = 42 healthy elderly volunteers and *n* = 42 patients with Mild Cognitive Impairment). They will receive fMRI neurofeedback twice (Intervention I and II), and behavioural and MRI data will be assessed before and after the intervention. The figure has been designed by the authors

The study will consist of seven appointments, five of which will take place in short succession with an additional two after 6 and 12 months (Fig. 1). We will first apply two baseline assessments (behavioural data followed by MRI data), two intervention sessions (i.e., the neurofeedback training), and one post-intervention (Fig. 1). We chose to split the two baseline assessments (i.e., behavioural data assessment and MR imaging) to avoid fatigue. All sessions will have approximately 1 week in-between and will last between 40 and 80 min. Six and 12 months after the post-intervention, we will apply follow-up assessments. Each of the assessments will be described in detail below, and an overview can be found in Table 1.

**Table 1:** Schedule of assessments conducted during the study

	Baseline I	Baseline II	Intervention I	Intervention II	Post-Intervention	Follow-up I	Follow-up II
Blood sample	X						X
*Questionnaires*							
Scale of Self-Efficacy	X						
Spontaneous use of Imagery	X						
Neo-Five Factor Inventory	X						
State-Trait Anxiety Inventory	X						
Profile of Mood States			X	X			
Positie and Negative Affect Schedule			X	X			
*Cognition*							
Montreal Cognitive Assessment^a^	X						
Cognitive Functions Dementia^b^	X						
Stroop	X						
Digit Span	X						
Verbal Learning and Memory Test		X	X	X	X	X	X
*MR Imaging*							
Structural MRI		X			X		X
Functional MRI		X			X		X
Neurofeedback			X	X			

^a^In healthy elderly volunteers

^b^In patients with Mild Cognitive Impairment

### Baseline behavioural assessment

At the first baseline assessment, we will acquire behavioural data (both with paper-and-pencil tasks and with Delta 1.4.2 on a tablet) as well as draw blood. We will start with drawing blood followed by cognitive screening with the MoCA in healthy volunteers or the Cognitive Functions Dementia battery (CFD) in patients with MCI due to AD. Although cognitive functions have already been evaluated during the diagnostic process in patients with MCI, we chose to apply another evaluation to account differences in time from diagnosis and study participation. Additionally, we will implement the Stroop task as well as the Digit Span task. After cognitive screening, we will employ several questionnaires, all of which have been validated in German: Spontaneous Use of Imagery Scale, Scale of Self-Efficacy, Neo Five-Factor Inventory, and State Trait Anxiety Inventory. The questionnaires will assess mental imagery, self-efficacy, personality traits, as well as anxiety all of which may influence the ability to regulate brain activity and/ or respond to feedback. Variables from the questionnaires will be used later for prediction analyses. We expect in particular that participants who score high on mental imagery as well as self-efficacy would benefit from the intervention. Likewise, those with high scores on openness and low scores on state/trait anxiety may be those who learn fast from feedback. Blood will be drawn to determine the apolipoprotein E (ApoE) genotype as well as the genetic variant of the brain derived neurotrophic factor (BDNF). The ApoE genotype seems to influence hippocampal activity [17, 18] and will, therefore, be included as a covariate in our statistical model. Likewise, we will include the BDNF genotype as it serves as a genetic modifier of brain plasticity and, thus, may influence the ability to learn from feedback. Finally, we will obtain Aβ42 as well as Aβ42/40 levels from blood using Simoa technology using methods established by the Neurochemistry Lab Amsterdam University Medical Centres [19]. Since hippocampal hyperactivity is particularly present in those with increased Aβ (and vice versa), the acquisition of Aβ is of specifal interest for this study.

### Baseline MRI

MRI data will be collected with a 32-channel head coil on a 3 Tesla Magnetom Prisma scanner (Siemens Medical Systems, Erlangen, Germany) at the Translational Imaging Center Bern. First, a T1-weighted structural image is obtained using a magnetization-prepared 2 rapid acquisition gradient echoes (MP2RAGE) sequence (176 slices, repetition time (TR) = 5000 ms, echo time (TE) = 2.98 ms, inversion time (TI) = 2500 ms, flip angle = 5°, matrix = 240 × 256, voxel size = 1 × 1 × 1 mm). Next, task-related blood oxygenation-level dependent data will be acquired using a T2*-weighted echo-planar imaging (EPI) sequence. One thousand eighty volumes will be collected during a pattern separation task (see below); each volume will consist of 56 slices with 2.5 mm thickness (flip angle = 30 °, TR = 1000 ms, TE = 37 ms, matrix size = 92 × 92, voxel size = 2.5 × 2.5 × 2.5 mm). Finally, non-hemodynamic resonant saturation effects will be assessed with a phase-cycled stimulus-induced rotary saturation approach [20]. A spin-lock radiofrequency pulse set to 120 Hz will be used to sensitize images to a frequency in the range of oscillating neuronal currents induced by epileptogenic tissue. Two T2*-weighted EPI sequences (300 volumes, slice thickness = 5 mm, skip = 10 mm, flip angle = 90 °, TR = 139.55 ms, TE = 29.12 ms, matrix size = 64 × 64, voxel size = 3.3 × 3.3 × 3.3 mm) will be acquired simultaneously. Later, T2* effects will be separated from rotary saturation effects by deconvolving spin-lock-off from spin-lock-on images.

### Pattern separation task

**Fig. 2:**
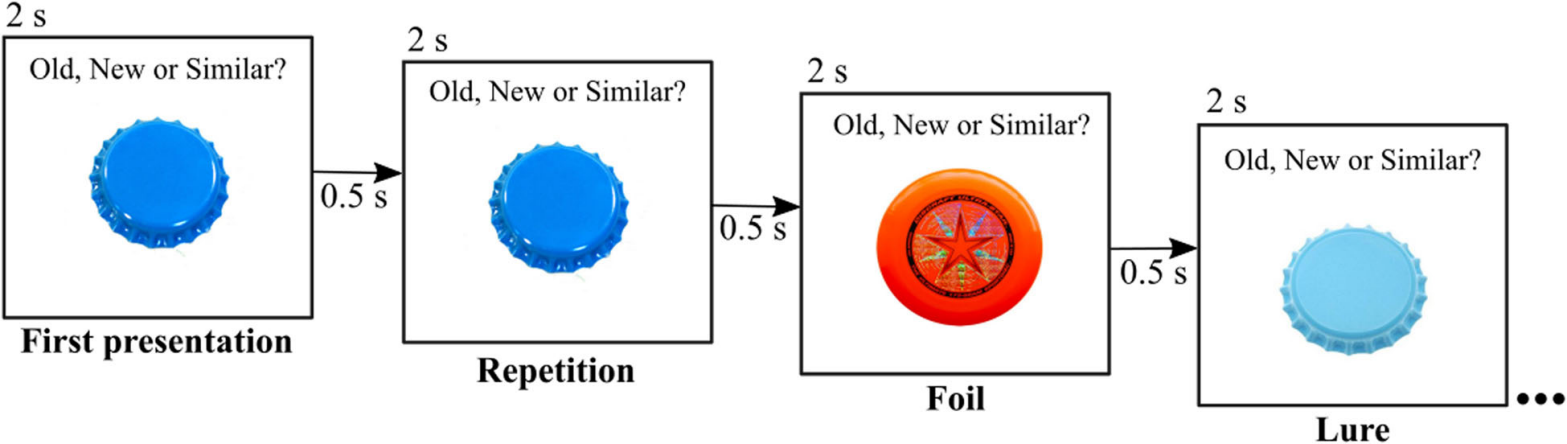
Pattern separation task applied during fMRI. For each run, 200 different objects will be randomly presented for 2 s (0.5 s inter-stimulus interval). The participants will need to decide whether an object is new, old, or similar. The figure has been designed by the authors with stimuli illustrations from https://github.com/celstark/MST

We chose to apply a pattern separation task, as hippocampal hyperactivity has been found particularly with this task in patients with MCI [7, 8]. The pattern separation task will be presented with PsychoPy (version 3.1 [21];). All participants will complete two runs of an explicit version of the task [22], during which coloured pictures of objects will be presented. These objects can be new (first presentations), repetitions of previously shown objects (repetitions), objects similar to those previously shown (lures), or new objects presented only once (foils; Fig. 2). Participants will need to categorize each object as either new, old, or similar. One run will include 200 stimuli, and each stimulus will be presented for 2 s with 0.5 s inter-stimulus interval. The 200 stimuli will include 40 foils, 40 repetitions, and 40 lures with an additional 40 of first presentations for repetitions and lures, respectively. The order of object presentation will be randomized for each participant. As in previous studies, the contrast of interest for the definition of baseline hippocampal activity will be lures > foils [7].

### Neurofeedback setup

**Fig. 3:**
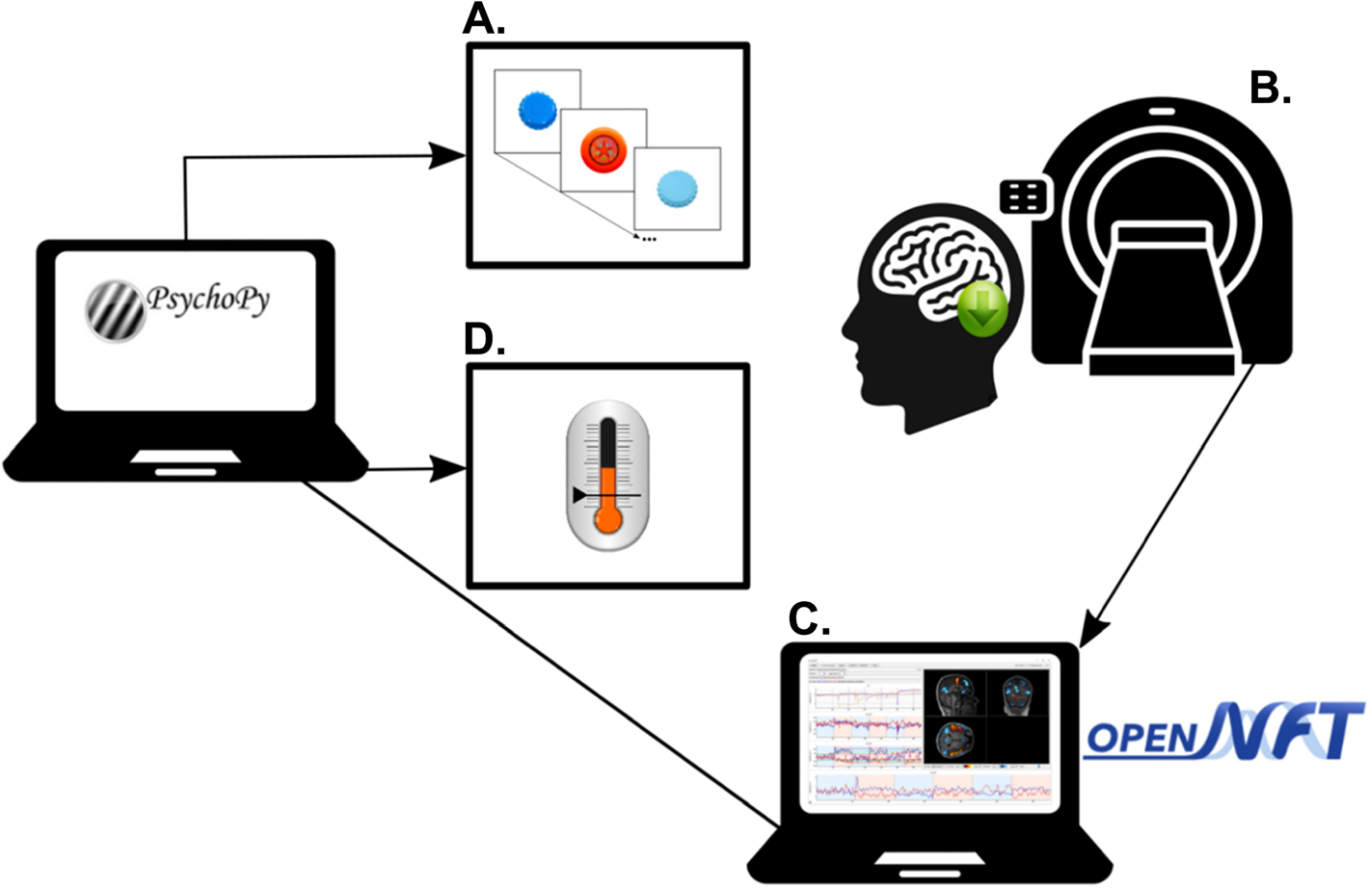
Neurofeedback setup. **a** The participants will see a slightly adapted version of the pattern separation task while they will need to downregulate activity in one of two regions of interest. **b** The functional brain activity in this region of interest will be measured continuously and sent to the laptop containing OpenNFT. **c** These data will be processed in real-time to estimate the percent signal change in the region of interest, which will be sent to the presentation laptop. **d** The value of the percent signal change will then be presented to the participants with a dynamic thermometer that changes colour from red (indicating increased activity) to green (indicating decreased activity). The figure has been designed by the authors with stimuli illustrations from https://github.com/celstark/MST and icons from www.flaticon.com

Approximately 1 week after baseline MRI, we will apply the neurofeedback training on 2 days with 1 week in-between. Each neurofeedback session will last about 60 min and will consist of five runs of training as well as two transfer runs (one prior to the feedback runs and another one directly following the feedback runs). The transfer run will assess the innate ability to regulate activity without feedback (first transfer run) or how much of the learned ability can be maintained without feedback (final transfer run). Each run (i.e., transfer or training) will consist of baseline blocks and regulation blocks (5:32 min in total) but only during training, feedback will be provided (Fig. 3). The feedback signal will be calculated with OpenNFT [23]. We will use the same EPI sequence as during baseline MRI. All DICOM images will be transferred directly from the imaging computer to a laptop running OpenNFT. OpenNFT will process these images in real-time (i.e., faster than image acquisition) and estimate whole-brain activation maps using an incremental general linear model (iGLM) algorithm. We will apply intermittent feedback (i.e., feedback presented after regulation as compared to during regulation), as this was shown to be more effective than continuous feedback in promoting self-modulation of brain activity [24]. The feedback signal will be estimated based on activity-levels in the respective region of interest (ROI) as the percent signal change (PSC) between baseline and regulation blocks. The PSC will be calculated as the average of the spatial-temporal data obtained from within the ROI. Further, OpenNFT will dynamically use the average of the highest and lowest activity time points of the acquired ROI data to estimate maximum and minimum limits of scaling used to calculate the final PSC value. The estimated feedback signal will be sent to the presentation computer and then presented to the participant using a thermometer icon (Fig. 3).

ROIs will be defined based on individual anatomical masks created with the individual T1-weighted images collected during baseline MRI. We will apply FreeSurfer (Linux-centos, v6.0.0, [25] to create individual ROIs and then co-register them to the motion correction file used for online realignment of the OpenNFT pre-processing pipeline.

### Neurofeedback session

**Fig. 4:**
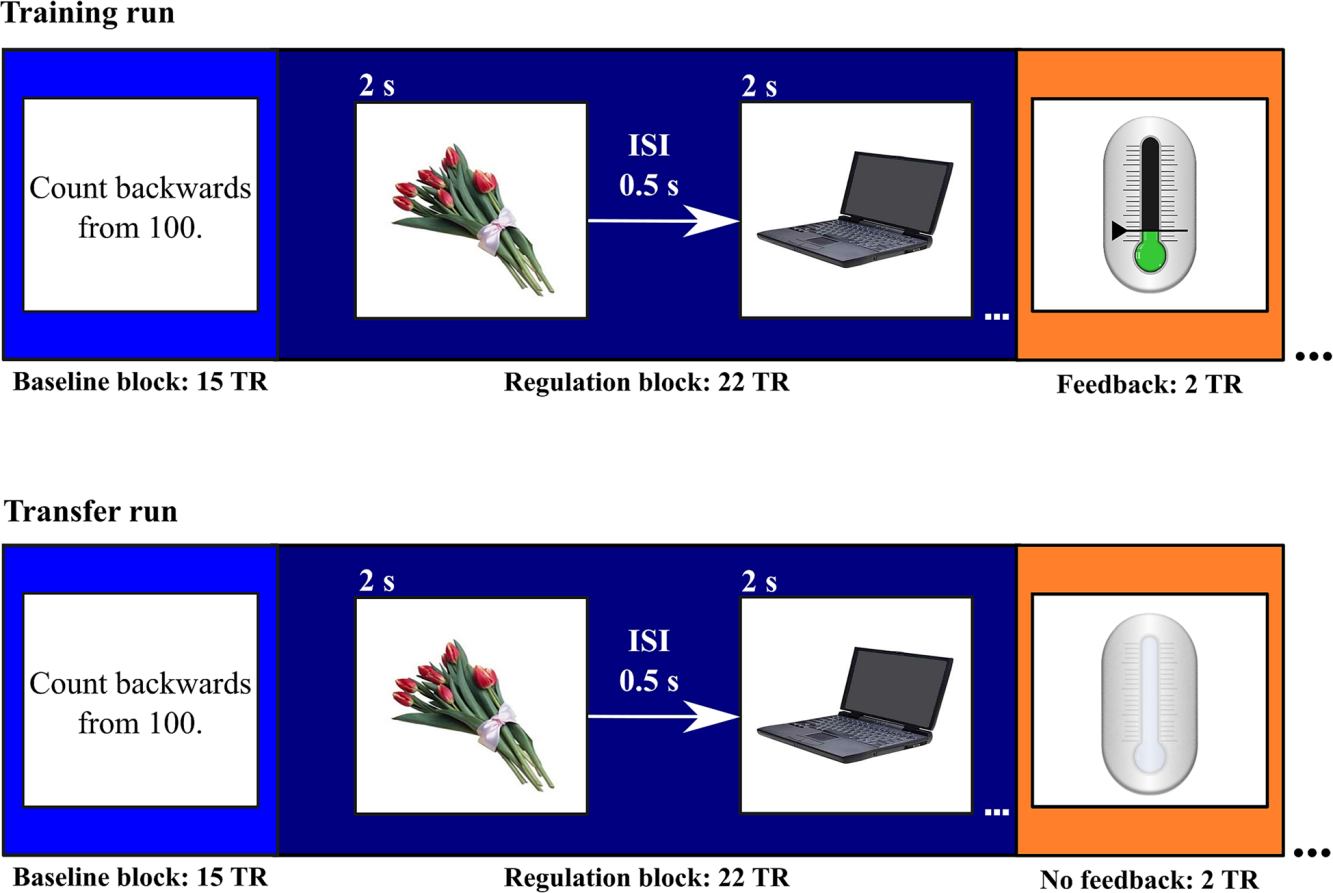
Neurofeedback session. Each run will include four repetitions of baseline and regulation blocks, as well as the feedback presented using a thermometer icon. The thermometer icon will be filled with colours ranging from red to green during training or will remain grey during transfer. TR: Repetition time (1 TR = 2 s). The figure has been designed by the authors with stimuli illustrations from https://github.com/celstark/MST

The neurofeedback task as well as the feedback will be presented with PsychoPy (version 3.1, [21]). For each session, we opted for a block design that includes four repetitions. During the baseline condition, participants will need to slowly count backwards from 100 for 30 s (Fig. 4). During the regulation condition, a slightly adapted version of the pattern separation task will be applied for 44 s. Again, the participants will be randomly presented with objects. These will include six foils, six lures and six first presentations of subsequent lures (i.e., 18 objects in total). Again, each object will be presented for 2 s with 0.5 s inter-stimulus interval (Fig. 4). In contrast to the fMRI task, however, the participants will not need to respond in any way to these images but rather try to downregulate activity. Previous research has shown that a response from participants is not required to induce hippocampal activity [26]. For the regulation of brain activity, we decided to use an implicit approach, which means that participants are told the goal would be to modify the thermometer icon until the green colour appears but we do not suggest explicit strategies for how to achieve this goal (i.e., they will need to try different mental strategies in order to find a strategy that works best for them [27, 28]. Feedback on the success of the regulation will be presented for 4 s. During the transfer runs, the participants will see a thermometer icon without feedback (Fig. 4).

Following the neurofeedback session, we will perform behavioural testing outside the scanner using a word list learning task (i.e., the VLMT [29];) that also probes memory functions. Afterwards, we will conduct debriefing of mental strategies. At the beginning and at the end of each session, we will apply the Profile of mood states (POMS [30];) and the Positive and Negative Affect Schedule (PANAS [31];) to assess whether the change in mood due to neurofeedback is similar for the control group and the experimental group.

### Post-intervention MRI

Approximately 1 week after the two neurofeedback sessions, participants will undergo post-intervention MRI. They will complete the fMRI pattern separation task that they have completed during baseline MRI (with different objects). In addition, we will again collect non-hemodynamic resonant saturation effects [20].

### Follow-up assessments

Approximately 6 and 12 months after the intervention, we will implement follow-up assessments. At the 6-months follow-up, we will only acquire behavioural data with the VLMT. At 12-months follow-up, we will apply the VLMT, draw blood again to obtain amyloid levels, and apply structural as well as functional MRI. For the fMRI task, we will again examine pattern separation performance (with different objects). In addition, participants will be examined using the MoCA (in healthy controls) or the CFD (in patients with MCI) for a second time.

### Statistical analyses

The primary outcome is the change in hippocampal activity as assessed with a pattern separation task during fMRI before and after both neurofeedback sessions. For the determination of intervention effects on hippocampal activity, we will compute repeated measures ANOVA with intervention (experimental vs. control group) and group (healthy volunteers vs. patients with MCI due to AD) as between-subject factors. We will include the genetic variants of ApoE and BDNF as covariates. Next, we are planning to compute regression analyses since we hypothesize that the reduction of hippocampal activity will be associated with memory performance change in the pattern separation task and the VLMT. As exploratory analyses, we will investigate whether any variable predicts successful neurofeedback; we will calculate the association between Aβ levels in blood and hippocampal activity; and we will identify the most successful downregulation strategies. For the prediction of successful neurofeedback, we will apply machine learning methods (e.g., cross-validated non-linear multivariate classification) to obtain the most important features.

### Sample size calculation

For the determination of sample size, we used G*power [32]. Since no study so far tried to downregulate hippocampal activity, we opted for the detection of at least a small effect (i.e., cohen’s *f* = 0.25). This would require an inclusion of *n* = 84 in total (that is, *n* = 21 in each group) for a repeated measures design with 4 groups and 4 measurements (i.e., baseline, post-intervention, follow-up 1, follow-up 2). We used the following criteria to calculate sample size: ANOVA with repeated measurements and between-within interactions, α err = 0.01, 1-β = 0.99, number of groups = 4, number of measurements = 4.

### Data management

All data will be entered by the study team and stored using Research Electronic Data Capture (REDCap), hosted by the Clinical Trials Unit (CTU) Bern. The study team is responsible for data management; data monitoring will be done by an independent researcher not involved in the study. We will record any spontaneously reported adverse events or other unintended effects of the intervention. An annual safety report will be submitted once a year to the local Ethics Committee. Study data will be stored on servers of Bern University, stripped of personal information of the participants. Unblinding will occur after all raw data have been transferred to the data analysis software. All computers will be password-protected and encrypted. At the end of the study, all personal data will be deleted. The procedures comply with Swiss data privacy laws.

## Discussion

This study will examine whether real-time fMRI neurofeedback can reduce hippocampal hyperactivity and whether the reduction will lead to an improvement in memory performance. This will extend former research showing that a pharmacological reduction of excess hippocampal activity in patients with MCI led to better memory performance using a pattern separation task [7]. In contrast to medication, real-time fMRI neurofeedback comes with considerably fewer risks or side effects [33]. In addition, it may give patients a sense of empowerment as they themselves tone down hyperactivity without having to ‘depend on’ taking medication. So far, the assessment of the effectiveness of downregulating the hippocampus requires using a set-up including an MR scanner. This study may provide insight into strategies that participants have successfully used to regulate hippocampal activity. It would be such strategies that may be included in future cognitive intervention studies. Should our study be successful, we will test whether other participants could use such strategies for themselves or whether each person will need to develop their own strategy. In the context of clinical use, only a few training sessions may produce long lasting effects [34, 35].

One aspect that may likely influence our results is the type of control group [28]. We opted for an active control rather than no feedback or yoked feedback; that is, feedback is given on the activity of a control region. In our view, only the comparison with an active control group can confirm that the results are specific to feedback from hippocampal activity [16]. However, differences between groups might still be related to differences in the perceived difficulty to regulate. We will account for this by evaluating how difficult participants thought it was to regulate.

Since we will gather blood samples to assess Aβ levels, we may also be able to replicate previous research in healthy elderly individuals, or patients with MCI, that reported higher levels of Aβ are associated with increased hippocampal activity [7, 9]. We will extend prior research by testing whether this association is also evident using blood-derived Aβ levels. In addition, blood samples will be used for the determination of the genetic variants of ApoE and BDNF. This will allow an exploration of the association of gene status with hippocampal hyperactivity, the ability to downregulate via neurofeedback, and the improvement in memory performance. Besides the primary goal of reducing hippocampal hyperactivity, we may also provide information as to whether successful neurofeedback can be predicted by variables extracted from questionnaires [27]. We expect that participants who score high on mental imagery as well as self-efficiency would benefit from the intervention. Likewise, those with high scores on openness and low scores on state/trait anxiety may be those who learn fast from feedback.

One limiting factor of our study might be the single-blind design. We will not inform the participants from which area they will receive feedback; yet, investigators will need to generate the ROI files and prepare the individual neurofeedback setup. Thus, they will not be blinded. In future studies, a double-blind protocol that could be implemented by separating investigators whot prepare the individual setup from investigators who instruct the participants would be desirable.

In sum, our study will provide insight into the efficacy of real-time fMRI neurofeedback to downregulate hippocampal hyperactivity in patients with MCI due to AD. It will further elucidate whether downregulation will lead to memory improvement. Data extracted from questionnaires may help to identify factors that predict successful neurofeedback.

## Data Availability

All data generated and/or analysed during the study will be made available from the corresponding author on request.

## Abbreviations

AD: Alzheimer’s disease
ANOVA: Analysis of Variance
ApoE: Apolipoprotein E
Aβ: Amyloid beta
BDNF: Brain derived neurotrophic factor
CERAD: Consortium to Establish a Registry for Alzheimer’s Disease
CFD: Cognitive Functions Dementia
CTU: Clinical Trials Unit
EPI: Echo Planar Imaging
fMRI: Functional magnetic resonance imaging
GDS: Geriatric Depression Scale
iGLM: Incremental general linear model
MCI: Mild Cognitive Impairment
MoCA: Montreal Cognitive Assessment
MP2RAGE: Magnetization-Prepared 2 Rapid Acquisition Gradient Echoes
PANAS: Positive and Negative Affect Schedule
POMS: Profile of mood states
PSC: Percent signal change
ROI: Region of interest
VLMT: Verbal Learning and Memory Task
